# [Expression of Concern] ZNF703 promotes tumor cell proliferation and invasion and predicts poor prognosis in patients with colorectal cancer

**DOI:** 10.3892/or.2026.9205

**Published:** 2026-09-30

**Authors:** Feng Ma, Lihong Bi, Gongli Yang, Mengnan Zhang, Cuiping Liu, Yingying Zhao, Yadong Wang, Jide Wang, Yang Bai, Yali Zhang

Oncol Rep 32: 1071–1077, 2014; DOI: 10.3892/or.2014.3313

Following the publication of the above article, a concerned reader has contacted the Editorial Office to draw attention to the fact that there appeared to be discrepancies between the numbers of patients reported with low- or high-ZNF703 expression in Fig. 3 on p. 1074: Fig. 3A reported 70 patients with low ZNF703 expression and 68 patients with high expression; however, the stage-stratified Kaplan-Meier curves showed the following: In Fig. 3B, stages I–IIIA: 57 low-expression and 20 high-expression patients, and in Fig. 3C, stages IIIB-IV: 20 low-expression and 41 high-expression patients (and so 70 vs. 68 patients in Fig. 3A, and 77 vs. 61 patients in Fig. 3B and C). In addition, the AJCC stage-specific counts reported in Table I also showed internal inconsistencies in terms of the overall numbers of low- and high-expression patients, and finally, an additional discrepancy was noted for the definition of the high expression group: The Materials and methods section stated that an immunohistochemical score of ≥6 was classified as high expression, whereas the Table I column heading defined high expression as ‘>6’. To clarify these apparent discrepancies, the authors should resolve the following: i) provide the actual numbers of low- and high-expression patients in each AJCC stage group; ii) clarify whether the cutoff used for the Kaplan-Meier analyses was ≥6 or >6; iii) provide the exact patient numbers used to generate Fig. 3A, B and C; and iv) clarify whether the counts, totals and percentages in Table I require correction. Finally, upon performing an independent analysis of the data in this paper in the Editorial Office, it came to light that, regarding the wound healing assay data shown in Fig. 6A on p. 1076, the ‘24 h / Control’ and ‘24 h / NC siRNA’ panels showed an overlapping section of data, such that data which were intended to show the results of experiments performed under different conditions had apparently been derived from the same original source.

The authors have been contacted by the Editorial Office to offer an explanation for the apparent anomalies in the description of the data, and the incorrect assembly of data in Fig. 6, in this paper, and we are awaiting their response. Owing to the fact that the Editorial Office has been made aware of this potential issue concerning the scientific integrity of this paper, we are issuing an Expression of Concern to notify readers of this potential problem while the Editorial Office continues to investigate this matter further.

